# Exercise Improves Immune Function, Antidepressive Response, and Sleep Quality in Patients with Chronic Primary Insomnia

**DOI:** 10.1155/2014/498961

**Published:** 2014-09-21

**Authors:** Giselle Soares Passos, Dalva Poyares, Marcos Gonçalves Santana, Alexandre Abílio de Souza Teixeira, Fábio Santos Lira, Shawn D. Youngstedt, Ronaldo Vagner Thomatieli dos Santos, Sergio Tufik, Marco Túlio de Mello

**Affiliations:** ^1^Universidade Federal de Goiás, Jataí, GO, Brazil; ^2^Universidade Federal de São Paulo, São Paulo, SP, Brazil; ^3^Universidade Estadual Paulista “Júlio de Mesquita Filho”, Presidente Prudente, SP, Brazil; ^4^College of Nursing and Health Innovation, and Exercise and Wellness, Arizona State University, Phoenix, AZ, USA; ^5^Phoenix VA Health Care System, Phoenix, AZ, USA; ^6^Universidade Federal de Minas Gerais, Belo Horizonte, MG, Brazil

## Abstract

The aim of this study was to evaluate the effects of moderate aerobic exercise training on sleep, depression, cortisol, and markers of immune function in patients with chronic primary insomnia. Twenty-one sedentary participants (16 women aged 44.7 ± 9 years) with chronic primary insomnia completed a 4-month intervention of moderate aerobic exercise. Compared with baseline, polysomnographic data showed improvements following exercise training. Also observed were reductions in depression symptoms and plasma cortisol. Immunologic assays revealed a significant increase in plasma apolipoprotein A (140.9 ± 22 to 151.2 ± 22 mg/dL) and decreases in CD4 (915.6 ± 361 to 789.6 ± 310 mm^3^) and CD8 (532.4 ± 259 to 435.7 ± 204 mm^3^). Decreases in cortisol were significantly correlated with increases in total sleep time (*r* = −0.51) and REM sleep (*r* = −0.52). In summary, long-term moderate aerobic exercise training improved sleep, reduced depression and cortisol, and promoted significant changes in immunologic variables.

## 1. Introduction

Chronic primary insomnia is a prevalent condition that affects 10–15% of the adult population, predominantly women [1]. Insomnia is particularly prevalent later in life, affecting approximately 12–25% of healthy seniors, and the prevalence is even higher among older adults with coexisting medical or psychiatric disorders [2].

Insomnia is often associated with occupational and psychosocial impairments, such as daytime fatigue, mood disturbances, cognitive deficits, and poor quality of life [3]. Some evidence suggests that sleep disturbances in depressed individuals are associated with impaired immune function [4, 5]; lower NK cell activity [6] and significantly lower levels of immune cells (CD3+, CD4+, and CD8+) [7] are associated with chronic insomnia.

Although the most common treatment for insomnia is pharmacotherapy, this type of treatment is associated with mortality [8] and other negative risk factors. Thus, nonpharmacological interventions have been suggested for the treatment of insomnia. Cognitive and behavioral therapies have been the most common nonpharmacological treatments [9, 10]. However, considering the high cost of maintaining these therapies, new nonpharmacological alternatives have been suggested, including regular exercise [11].

Acute [12] and chronic aerobic exercise [13–15] studies have demonstrated significant reductions in insomnia. Among the positive effects of aerobic exercise on health is the promotion of immune function [16]. The objective of the present study was to evaluate the effects of aerobic exercise on sleep, depression symptoms, immune function, and cortisol levels among patients with chronic primary insomnia.

## 2. Methods

The study was approved by the University Human Research Ethics Committee and conformed to the principles outlined in the Declaration of Helsinki. The participants were recruited through advertisements in newspapers, magazines, and radio shows.

### 2.1. Screening

Interested prospective participants contacted the laboratory and were initially screened in a phone interview. The initial inclusion criteria included being between 30 and 55 years old; having insomnia complaints for longer than 6 months; and having at least one complaint of daytime impairment resulting from insomnia (i.e., mood, cognition, or perception of fatigue). The initial exclusion criteria included the use of medication or psychotherapeutic drugs for insomnia or other psychiatric disorders, shiftwork, and exercising ≥1 day per week.

Prospective participants who passed the phone screen were invited to the laboratory for further orientation to the study and screening. During the visit, the prospective participants signed a written informed consent form approved by the ethics committee.

Further medical screening included establishing a clinical diagnosis of primary insomnia according to the DSM-IV [17] and the American Academy of Sleep Medicine [18] for a period longer than 6 months. Additional exclusion criteria included blood tests contraindicating participation in exercise, having major depression (i.e., a Beck Depression Inventory score >20; [19], having an apnea-hypopnea index (AHI) >15, having a periodic leg movement index (PLMI) >15, and being a shift worker or all-night worker).

The participants received a clinical consultation and an electrocardiogram at rest and under maximal stress during cardiopulmonary exercise testing (CPET). The CPET was performed on a treadmill (Life Fitness 9500 HR) with an initial velocity of 4 km/h, increasing the speed by 0.5 km/h each minute until voluntary exhaustion. Breath-by-breath assessments were collected using a metabolic system (Quark PFT4, Rome, Italy). The highest $\dot{\text{V}}$O_2_ value obtained during the last 20 seconds of the test was considered to be the peak oxygen uptake ($\dot{\text{V}}$O_2_ peak). The ventilatory threshold (VT_1_) was estimated by inspecting the inflection point of $\dot{\text{V}}$CO_2_ with respect to $\dot{\text{V}}$O_2_ (modified V-slope) [20, 21]. A certificate attesting to the patient's ability to participate in the exercise protocol was provided by his or her physician and served as the final requirement for participation in the study.

### 2.2. Exercise Training

Following screening, the participants performed the exercise training regimen for 4 months. Exercise was performed 3 days per week for 50 continuous minutes per bout at the VT_1_ [20, 21], established during the CPET. The timing of the exercise was randomized to occur in the morning (10:00 ± 1 h) or in the evening (18:00 ± 1 h).

### 2.3. Polysomnographic Measures

The participants underwent polysomnographic (PSG) recording at the Sleep Institute before and after the exercise training. For the preintervention assessment, PSG recording was performed over 2 nights. The first night served as an adaption night; 48 hr later, the participants returned for another night of PSG recording. The posttraining PSG assessment occurred at least 30 hours after the last exercise session. The participants arrived at the sleep laboratory at 21:00; the PSG recording started and finished according to each volunteer's habitual sleep schedule. The digital system EMBLA was used (EMBLA S7000, Embla Systems Inc., CO, USA). The measured variables included total sleep time (TST), sleep efficiency (the ratio between total sleep time and total recorded time multiplied by 100), sleep onset latency, waking after sleep onset, arousals, sleep stages (I, II, III, and IV of NREM sleep and REM sleep), and the latencies for each sleep stage, including Stage 1, Stage 2, Stages 3 and 4, and REM. The apnea-hypopnea index (AHI) and periodic leg movements (PLM) were also measured. Two researchers who were blinded to the study design performed the staging and analyzed the PSG events using international criteria [22–24].

### 2.4. Questionnaires and Blood Draw

Approximately twenty-four hours after the PSG assessment, the subjects arrived in the laboratory at 9 a.m. They completed the Pittsburgh Sleep Quality Index [25] and Beck Depression Inventory [19]. At approximately 10-11 a.m. preprandial venous blood was drawn from the medial cubital vein.

### 2.5. Immunologic Assays

#### 2.5.1. Immunological Measures

Total leukocyte and subgroup cell counts were determined using an automated hematology analyzer (Advia 120, Siemens). CD4 and CD8 populations were determined by flow cytometry using the CD3/CD4 or CD3/CD8 expression in the cell surface (FACSCalibur, Becton Dickison, San Jose, CA, USA).

#### 2.5.2. Cortisol and Apolipoprotein

Plasma cortisol concentrations were determined using a chemiluminescence immunoassay (Unicell DXI, Beckman Coulter, Fullerton, CA, USA), CV% within assay: 4.4. CRP was determined by nephelometry (Image 800, Beckman Coulter), CV% within assay: 5.0. Apolipoprotein A (ApoA) and apolipoprotein B (ApoB) were measured using nephelometry (Image 800, Beckman Colter), CV% within assay: 4.0.

### 2.6. Statistical Analysis

The program STATISTICA (Statsoft, Inc., version 7.0) was used for data analysis. We tested normality by Shapiro Wilk's test. For normally distributed data we used Student's paired *t*-test (to compare the pre- and postexercise measures) and for data that violated normality we used Wilcoxon matched pairs test. Spearman's correlation coefficient was performed to assess associations between immune system variables and sleep data (after exercise-baseline means). The data are expressed as the mean ± SD. The level of statistical significance was set at *P* < .05.

## 3. Results

### 3.1. Recruitment, Participant Attrition, and Adherence to the Exercise Protocol

**Figure 1:**
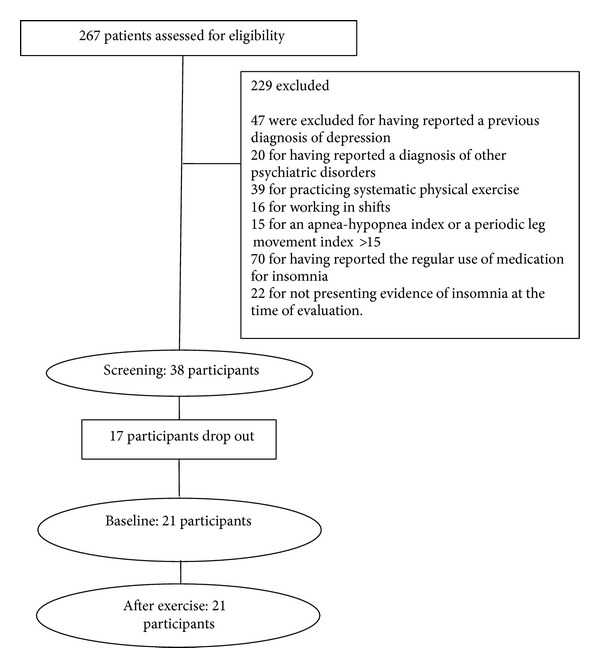
Participant flowchart.

Two hundred sixty-seven people contacted the researchers by telephone or email with interest in participating in the study. Of this total, 229 were excluded because they did not meet the inclusion criteria (Figure 1). Thirty-eight participants (29 women) passed the initial screening. However, before beginning the exercise training protocol, 3 men and 5 women withdrew from the study during the preintervention period.

Thus, the exercise protocol began with a sample of 30 participants. However during the first month of the study 11 participants drop out. Of these, 9 (30%) dropped out because of problems with the program schedule, which could not be changed during the study. As a result, the final sample size was 21. These 21 participants exhibited good adherence to the study protocol (>90%) based on their class attendance and the percentage of the exercise time they spent within the prescribed intensity (90%).

### 3.2. Physiological Parameters

Significant increase in speed in VT_1_ (5.3 ± 1 to 6.1 ± 1 km/h, *P* < .001) was found after exercise training (Table 1).

### 3.3. Polysomnography

Compared with baseline, significant reductions were observed in sleep onset latency (approximately 14 min, *P* < .01), wake after sleep onset (approximately 15 min, *P* = .03), and REM latency (approximately 24 min *t*(29) = 0.01), and significant increases were found in total sleep time (approximately 24 min, *t*(29) = 0.02) and sleep efficiency (approximately 7%, *t*(29) = 0.002) in the postexercise evaluation. The percentage of REM sleep increased significantly (2.5%, *t*(29) = 0.04), and the latency of Stage 2 (approximately 13 min, *P* = .01) and latency of Stages 3 and 4 decreased significantly (approximately 22 min, *P* = .02) (Table 2).

### 3.4. Subjective Scales

Subjective sleep rating on the Pittsburgh Sleep Quality Index (PSQI) revealed significant improvement on the subjective sleep duration (approximately 1 h, *t*(29) < 0.001) and a significant reduction in sleep onset latency (24 min, *P* = .005) following exercise training compared with preintervention. The global score on the PSQI was reduced significantly (approximately 40%, *t*(29)<.0001) after the exercise intervention. The Beck Depression Inventory score decreased significantly after exercise training (approximately 30%, *t*(29) = 0.04) (Table 3).

### 3.5. Immune Profile

We observed a reduction in plasma cortisol concentrations after exercise training (13.7 ± 5 to 10.6 ± 4 *μ*g/dL, *P* = .02). Plasma apolipoprotein A increased (139.2 ± 26 to 153.9 ± 24 mg/dL, *P* < .001), and erythrocytes (4.8 ± 0.4 to 4.6 ± 0.4 10^6^/mm^3^, *P* = .02), total leukocytes (6.8 ± 1 to 5.7 ± 2 mil/mm^3^, *P* < .001), monocytes (0.39 ± 0.2 to 0.26 ± 0.1 mil/mm^3^, *P* < .001), and typical lymphocytes (2.1 ± 0.7 to 1.7 ± 0.5 mil/mm^3^, *P* < .001) decreased significantly after the intervention. We also observed significant decreases in the CD4 (938.8 ± 381 to 778.9 ± 324 mm^3^, *P* < .001) and CD8 counts (572.2 ± 265 to 434.1 ± 197 mm^3^, *P* < .0010) (Table 4).

### 3.6. Correlation of Immunity and Cortisol with Sleep Data

Significant correlations between the decrease in cortisol and increases in polysomnographic measures of total sleep time (*r* = −0.50, *P* < .05) and REM sleep (*r* = −0.47, *P* < .05) were observed. There were no significant correlations between changes in cortisol or immunity measures and changes in subjective or objective sleep data.

## 4. Discussion

The purpose of this study was to investigate the effects of exercise training on sleep, depression symptoms, immunity, and cortisol levels of patients with chronic primary insomnia. The results showed significant improvements in objective and subjective sleep and a reduction in depression symptoms after exercise training. Moreover, there were decreases in plasma cortisol levels and changes in the immunologic parameters, which were correlated with some of the sleep improvements. The results are consistent with previous research showing the benefits of exercise training on sleep and depression symptoms among patients with chronic primary insomnia [12, 14, 15].

It has been suggested that persistent hyperarousal at autonomous, emotional, cognitive, or neurobiological levels may be the decisive factor in the development and persistence of chronic primary insomnia [26–28]. This pathway could be attenuated by well-established anxiety-reducing effects of exercise [29].

Among the factors discussed with regard to chronic primary insomnia have been increases in the production of cortisol [26] and an increase in immune system indicators, such as interleukin-6, during the daytime [30] and nighttime [31].

Exercise training might be a particularly helpful nonpharmacological stimulus for controlling metabolic regulation and immune function and improving sleep [32]. There is abundant evidence that exercise promotes immune function [33–36]. We recently demonstrated that acute and chronic exercise (aerobic and/or resistance exercise) improves the metabolic profile and quality of sleep in elderly people [37, 38]. In the present study, our data showed that chronic exercise also induced positive effects on the immune system and sleep in patients with chronic primary insomnia.

We found a significant reduction in CD4 and CD8 counts and a trend toward a reduction in the ratio of CD4 : CD8. Similar results were found by Unal et al. [39]: the authors observed a decrease in CD4 cells in sedentary male university students after 8 weeks of moderate exercise training. The minor changes in CD4 and CD8 counts in the present study were associated with the anti-inflammatory response induced by chronic exercise [40].

We also found a parallel decrease in monocytes and basophils after exercise. These data corroborate those found by Kennedy et al. [41] and Michishita et al. [42] in animals and humans, respectively.

ApoA is a surrogate marker for HDL-C and has been measured in recent prospective epidemiological studies [43]. Increasing the HDL-C level by 1 mg/dL may reduce the risk of cardiovascular disease by 2% to 3% [44]. This result is consistent with the concept of the beneficial role of HDL-C, which is based on free cholesterol efflux from macrophages out of the vessel wall. Of these, the ABCA1 transporter system is the most efficient, responsible for over 50% of cholesterol efflux from macrophages to poorly lipidated ApoA. Mature HDL-C also transfers esterified cholesterol to other lipoproteins via the enzyme cholesterol ester transporter protein (CETP), increasing the efficiency of the system [45].

There were some notable limitations of the present study. First, the sample size was small, which resulted in limited statistical power.

Second, without a control treatment, it cannot be ascertained whether the results were influenced by a number of potential confounding variables associated with participating in the study, including the demand or expectancy effects associated with volunteering for an exercise study for insomnia, the social interaction between the participants and staff, and spontaneous remission. Moreover, the exercise regimen was performed in front of large windows with a beautiful view of the city, so the results might be partially explained by increased exposure to bright light, which can have sleep- and mood-promoting effects. Also, because interim data were not assessed, it is unclear how long the intervention must be to produce these effects. Research staff also were not blinded to treatment, which could have led to further biases towards positive results.

Another limitation was the large age range of participants (ages 30–55 y), which could have contributed to greater variability in sleep. However, since this was a within-subjects design, this was less of a confound.

Notwithstanding these limitations, the results suggest that long-term moderate-intensity aerobic exercise improves the immunological profile, increases apolipoprotein A, and promotes anti-inflammatory and antiatherogenic changes. The data can provide some insight into sleep quality in patients with chronic primary insomnia, and perhaps some of the improvement in immunology and inflammation caused by the improvements in sleep after exercise could improve sleep quality and minimize some of the impairment associated with chronic primary insomnia.

## Figures and Tables

**Table 1:** Physiological parameters.

Variable	Baseline (*N* = 21)	After exercise (*N* = 21)	*P*
BMI (kg/m^2^)	24.7 ± 5	24.6 ± 5	ns
VO_2peak_ (mL/kg/min)	28.4 ± 7	30.3 ± 6	ns
Speed VT_1_ (km/h)	5.3 ± 1	6.1 ± 1	<.001

Wilcoxon matched pairs test, significant results, *P* < .05; data are expressed as mean ± SD. VO_2peak_: peak oxygen consumption; VT_1_: first ventilatory threshold.

**Table 2:** Polysomnography variables on the baseline and after exercise.

Variable	Baseline (*N* = 21)	After exercise (*N* = 21)	*P*
TST (hours)	5.7 ± 0.8	6.1 ± 0.5	.02
∗SOL (min)	22.5 ± 23	8.6 ± 8	<.001
LREM (min)	100.4 ± 38	76.0 ± 43	.01
SE (%)	82.2 ± 10	88.9 ± 8	.006
∗WASO (min)	53.2 ± 49	38.2 ± 25	.03
Arousals (events/hour)	17.8 ± 23	15.6 ± 13	ns
Stage 1 (%)	3.9 ± 2	3.9 ± 3	ns
∗Stage 2 (%)	56.7 ± 7	54.5 ± 8	ns
Stage 3 (%)	4.6 ± 2	5.1 ± 2	ns
Stage 4 (%)	15.3 ± 5	14.5 ± 6	ns
REM sleep (%)	19.4 ± 6	21.9 ± 6	.04
∗Latency of Stage 1 (min)	80.6 ± 89	38. 9 ± 77	ns
∗Latency of Stage 2 (min)	23.6 ± 23	10.5 ± 11	.01
∗Latency of Stages 3 and 4 (min)	55.6 ± 51	33.7 ± 18	.04
∗AHI (events/hour)	9.7 ± 9	8.1 ± 9	ns
∗PLM (events/hour)	2.0 ± 4	2.2 ± 5	ns

Paired Student's *t*-test or ∗Wilcoxon matched pairs test, significant results, *P* < .05; data are expressed as mean ± SD. TST: total sleep time; SE: sleep efficiency; SOL: sleep onset latency; REM: rapid eye movements; LREM: REM sleep latency; TWT: total wake time; WASO: wake time after sleep onset; AHI: apnea hypopnea index; PLM: periodic leg movements.

**Table 3:** Subjective scales on baseline and postexercise evaluations.

Variable	Baseline (*N* = 21)	After exercise (*N* = 21)	*P*
PSQI (score)	11.2 ± 3	6.8 ± 2	.001
∗PSQI-SOL (min)	46.5 ± 22	22.0 ± 10^#^	.005
PSQI-SE (%)	75.3 ± 29	81.8 ± 17	Ns
PSQI-TST (h)	4.6 ± 2	6.3 ± 2	.001
BDI (score)	9.3 ± 3	6.5 ± 5	.04

Paired Student's *t*-test or ∗Wilcoxon matched pairs test, significant results, *P* < .05; ^#^ *n* = 20; data are expressed as mean ± SD. PSQI: Pittsburgh Sleep Quality Index; BDI: Beck Depression Inventory.

**Table 4:** Cortisol and immune profile of volunteers on the baseline and after exercise.

Variable	Baseline (*N* = 21)	After exercise (*N* = 21)	*P*
Cortisol (*μ*g/dL)	13.7 ± 5	10.6 ± 4	.02
CD4 (mm^3^)	938.8 ± 381	778.9 ± 324	<.001
CD8 (mm^3^)	572.2 ± 265	434.1 ± 197	<.001
CD4 : CD8	1.77 ± 0.6	1.9 ± 0.7	ns
C-reactive protein (mg/dL)	0.22 ± 0.2	0.19 ± 0.1	ns
Apolipoprotein A (mg/dL)	139.2 ± 26	153.9 ± 24	<.001
Apolipoprotein B (mg/dL)	98.1 ± 26	94.4 ± 21	ns
Immunoglobulin A (mg/dL)	259.6 ± 123	262.6 ± 103	ns
Hematocrit (%)	42.0 ± 4	41.7 ± 4	ns
Erythrocytes (10^6^/mm^3^)	4.8 ± 0.4	4.6 ± 0.4	.02
Hemoglobin (g/dL)	14.0 ± 1	13.7 ± 1	ns
Total leukocytes (mil/mm^3^)	6.8 ± 1	5.7 ± 2	<.001
Neutrophils (mil/mm^3^)	4.1 ± 1	3.6 ± 1	ns
Monocytes (mil/mm^3^)	0.39 ± 0.2	0.26 ± 0.1	<.001
Typical lymphocytes (mil/mm^3^)	2.1 ± 0.7	1.7 ± 0.5	<.001
Eosinophils (mil/mm^3^)	0.16 ± 0.1	0.14 ± 0.1	ns
Basophils (mil/mm^3^)	0.05 ± 0.02	0.04 ± 0.02	ns
Platelets (mil/mm^3^)	271.6 ± 66	276 ± 63	ns

Wilcoxon matched pairs test, significant results, *P* < .05; data are expressed as mean ± SD.
